# Impact and effectiveness of 13-valent pneumococcal conjugate vaccine on population incidence of vaccine and non-vaccine serotype invasive pneumococcal disease in Blantyre, Malawi, 2006–18: prospective observational time-series and case-control studies

**DOI:** 10.1016/S2214-109X(21)00165-0

**Published:** 2021-06-15

**Authors:** Naor Bar-Zeev, Todd D Swarthout, Dean B Everett, Maaike Alaerts, Jacquline Msefula, Comfort Brown, Sithembile Bilima, Jane Mallewa, Carina King, Anne von Gottberg, Jennifer R Verani, Cynthia G Whitney, Charles Mwansambo, Stephen B Gordon, Nigel A Cunliffe, Neil French, Robert S Heyderman

**Affiliations:** a Malawi–Liverpool–Wellcome Trust Clinical Research Programme, College of Medicine, University of Malawi, Blantyre, Malawi; b Center for Global Vaccine Research, University of Liverpool, Liverpool, UK; c NIHR Health Protection Research Unit in Gastrointestinal Infections, University of Liverpool, Liverpool, UK; d International Vaccine Access Center, Department of International Health, Bloomberg School of Public Health, Johns Hopkins University, Baltimore, MD, USA; e NIHR Global Health Research Unit on Mucosal Pathogens, Research Department of Infection, Division of Infection and Immunity, University College London, London, UK; f The Queens Medical Research Institute, University of Edinburgh, Edinburgh, UK; g Cardiogenetics Research Group, Center of Medical Genetics, University of Antwerp and Antwerp University Hospital, Antwerp, Belgium; h College of Medicine, University of Malawi, Blantyre, Malawi; i Department of Global Public Health, Karolinska Institutet, Stockholm, Sweden; j Centre for Respiratory Diseases and Meningitis, National Institute for Communicable Diseases, Division of the National Health Laboratory Service, Johannesburg, South Africa; k School of Pathology, University of the Witwatersrand, Johannesburg, South Africa; l National Center for Immunization and Respiratory Diseases, Centers for Disease Control & Prevention, Atlanta, GA, USA; m Ministry of Health, Lilongwe, Malawi; n Liverpool School of Tropical Medicine, Liverpool, UK

## Abstract

**Background:**

The population impact of pneumococcal conjugate vaccines (PCVs) depends on direct and indirect protection. Following Malawi's introduction of the 13-valent PCV (PCV13) in 2011, we examined its impact on vaccine and non-vaccine serotype invasive pneumococcal disease among vaccine-eligible-age and vaccine-ineligible-age children and adults.

**Methods:**

We did a prospective observational time-series analysis and a case-control study. We used data from between Jan 1, 2006, and Dec 31, 2018, from laboratory-based surveillance at a government hospital in Malawi. This period included 6 years before and 7 years after introduction of PCV13. By use of negative-binomial regression, we evaluated secular trend-adjusted incidence rate ratio (IRR) in vaccine serotype and non-vaccine serotype invasive pneumococcal disease before and after introduction of PCV. We compared predicted counterfactual incidence in hypothetical absence of vaccine with empirically observed incidence following vaccine introduction. The case-control study assessed vaccine effectiveness, comparing PCV uptake among cases of vaccine-eligible-age invasive pneumococcal disease versus matched community controls.

**Findings:**

Surveillance covered 10 281 476 person-years of observation, with 140 498 blood and 63 291 cerebrospinal fluid cultures. A reduction in total (vaccine serotype plus non-vaccine serotype) invasive pneumococcal disease incidence preceded introduction of PCV: 19% (IRR 0·81, 95% CI 0·74 to 0·88, p<0·0001) among infants (<1 year old), 14% (0·86, 0·80 to 0·93, p<0·0001) among children aged 1–4 years, and 8% (0·92, 0·83 to 1·01, p=0·084) among adolescents and adults (≥15 years old). Among children aged 5–14 years there was a 2% increase in total invasive pneumococcal disease (1·02, 0·93 to 1·11, p=0·72). Compared with the counterfactually predicted incidence, incidence of post-PCV13 vaccine serotype invasive pneumococcal disease was 74% (95% CI 70 to 78) lower among children aged 1–4 years and 79% (76 to 83) lower among children aged 5–14 years, but only 38% (37 to 40) lower among infants and 47% (44 to 51) lower among adolescents and adults. Although non-vaccine serotype invasive pneumococcal disease has increased in incidence since 2015, observed incidence remains low. The case-control study (19 cases and 76 controls) showed vaccine effectiveness against vaccine serotype invasive pneumococcal disease of 80·7% (–73·7 to 97·9).

**Interpretation:**

In a high-mortality, high-HIV-prevalence setting in Africa, there were significant pre-vaccine reductions in the incidence of invasive pneumococcal disease. 7 years after PCV introduction, although vaccine-attributable impact among vaccine-eligible-age children was significant, indirect effects benefitting unvaccinated infants and adults were not. Policy decisions should consider multiple alternative strategies for reducing disease burden, including targeted vaccination outside infant Expanded Programme of Immunization to benefit vulnerable populations.

**Funding:**

Bill & Melinda Gates Foundation, Wellcome Trust, and National Institute for Health Research.

## Introduction

*Streptococcus pneumoniae* is estimated to be responsible for more than 318 000 deaths per year (uncertainty ratio 207 000–395 000) in children aged 1–59 months worldwide, with the highest mortality burden in Africa.^1^ *S pneumoniae* has almost 100 serotypes and is a common coloniser of the human nasopharynx, particularly in young children and resource-poor and HIV-affected populations.^2^ The contribution to adult mortality is poorly quantified but large, particularly in older adults and high-risk groups such as individuals with HIV infection. Although most carriers are asymptomatic, pneumococcal colonisation is a prerequisite for transmission and might also lead to the development of disease, including pneumonia, meningitis, and sepsis.

**Research in context**

**Evidence before this study**

The PCV (pneumococcal conjugate vaccine) Review of Impact Evidence (PRIME) systematic review included 14 databases with data from Jan 1, 2010, to Dec 31, 2016, and concluded that PCV had had an impact on multiple pneumococcal endpoints. However, the existence of few robust data from sub-Saharan Africa was highlighted as a gap in knowledge, with only seven of the 44 studies included in the invasive pneumococcal disease analysis being from Africa. Extrapolating results of studies from high-income countries to sub-Saharan African countries is problematic. On April 1, 2020, We implemented a targeted literature review strategy using PubMed, restricting the search to Africa, and incorporating the PRIME search strategy: pneumococc* OR “strep* pneumo*” OR “s. pneumo*” OR streptococc* AND “invasive pneumococcal disease” OR “IPD” OR invasive bacterial disease AND “pneumococcal vaccines” OR “vaccines, conjugate” OR “streptococcal vaccines” AND vaccin* OR immuniz* OR immunis*. We did not incorporate any language or date restrictions. After reviewing 119 articles, 13 met the inclusion criteria for reporting vaccine impact on invasive pneumococcal disease surveillance data. Although reports varied in quality, with most reporting administrative data on hospital admissions, there was consistent evidence of PCV impact on reducing invasive pneumococcal disease. The overall reduction in incidence of invasive pneumococcal disease ranged from 31% to 85%. Invasive pneumococcal diseases caused by vaccine serotypes also decreased incidence of substantially, ranging from 35% to 91%. A common reported limitation was a short pre-PCV13 (13-valent PCV) observation period, which can introduce bias as a consequence of non-vaccine-associated trends in disease burden—a major issue in Africa in the context of substantial improvements in child health in the past 20 years.

**Added value of this study**

We did a large-scale analysis of PCV13 impact on vaccine serotype and non-vaccine serotype invasive pneumococcal disease incidence based on 13 years of surveillance (10 281 476 person-years at risk), including 6 years before and 7 years after Malawi's 2011 introduction of PCV13 into its national immunisation programme. The ongoing invasive pneumococcal disease surveillance has benefited from stable and consistent laboratory methods. We report a long-standing reduction in invasive pneumococcal disease incidence over the 13 years of analysis. These findings address knowledge gaps highlighted in the 2017 WHO technical expert consultation, with clear evidence of considerable PCV-induced reduction of the incidence of vaccine serotype invasive pneumococcal disease among children but a lesser indirect effect among infants younger than 1 year old and individuals aged 15 years or older. Our findings underscore the need for a long observation period to understand the impact of PCV in a dynamic context.

**Implications of all the available evidence**

The scale of herd protection after implementation of PCV will depend on several factors including vaccine serotype carriage prevalence, the proportion of disease caused by vaccine serotypes, force of infection, and population structure and mixing. This study showed a direct reduction in the incidence of invasive pneumococcal disease among children following introduction of PCV13, accelerating the pre-existing reductions in incidence. However, by contrast with high-income countries, and despite high vaccination coverage, indirect protection among infants and adults was more modest. Control of pneumonia and invasive pneumococcal disease in young adults with HIV remains necessary in Africa. Improving herd protection with alternative vaccine strategies (including alternative schedules) needs evaluation as well as reconsideration of a policy to vaccinate high-risk adolescents and adults.

Despite evidence of residual vaccine-serotype carriage across sub-Saharan Africa, including Malawi,^3^, ^4^ vaccine trials and post-routine-introduction studies have shown the substantial direct protection of pneumococcal conjugate vaccines (PCVs) against invasive pneumococcal disease incidence among age-eligible children.^5^, ^6^ In many settings, PCVs have also been associated with herd protection with resulting reductions in the incidence of invasive pneumococcal disease among vaccine-ineligible groups through indirect means, presumably through their impact on nasopharyngeal carriage and subsequent transmission to unvaccinated individuals.^6^, ^7^, ^8^ In high-income countries, groups benefiting from the indirect effects of infant vaccination include the very young,^9^ older adults,^10^ and HIV-infected adults.^11^ However, reported replacement disease, with an absolute increase in the incidence of non-vaccine serotype invasive pneumococcal disease, has eroded the impact seen initially after PCV introduction.^5^, ^12^

In Malawi—a southern African country consistently ranked by the World Bank in the lowest income category—invasive pneumococcal disease prevalence by age closely mirrors HIV prevalence. HIV-infected adults remain at high risk of invasive pneumococcal disease, so they would probably benefit from PCV vaccination.^13^, ^14^ In 2015, mean HIV prevalence in Malawi was 8·8%, reaching 19·8% among urban women.^15^ Free antiretroviral therapy has been provided in Malawi since 2004 through the support of the Global Fund. Life expectancy among HIV-infected people has increased markedly since antiretroviral therapy was first introduced in 2004 and made widely available in 2006.^14^, ^16^ In 2011, Malawi adopted Option B+, whereby all HIV-infected pregnant or breastfeeding women commence lifelong antiretroviral therapy regardless of clinical or immunological stage, dramatically reducing mother-to-child transmission. On Nov 12, 2011, Malawi (previously PCV-naive) introduced 13-valent PCV (PCV13) as part of the national Expanded Programme of Immunization, using a 3+0 schedule (three primary doses [6, 10, and 14 weeks of age] with no booster dose). A catch-up vaccination campaign included infants younger than 1 year of age at the date of first dose, receiving three doses at 1-month intervals. Field studies in Malawi have reported high PCV13 uptake of 90–95%,^17^ similar to the 92% PCV13 coverage reported by WHO and UNICEF.^18^ Furthermore, we have reported good adherence to the dosing schedule in Malawi, with the median age at first PCV being 6·3 weeks (IQR 4·9–8·1), 11·2 weeks (9·1–14·1) at second dose, and 16·4 weeks (11·4–19·5) at third dose.^4^

In Malawi, we have demonstrated the early onset of pneumococcal colonisation,^19^ a high prevalence of residual vaccine serotype carriage among children since introduction of PCV13 in 2011,^4^ and persistent high prevalence of pneumococcal carriage among HIV-infected adults receiving antiretroviral therapy.^20^, ^21^ We also projected that the 10-year vaccine serotype carriage reduction among 0–9-year-old children will be lower than observed in other settings, mainly driven by a high local force of infection (the rate by which a certain age group of susceptible individuals is infected).^22^ Here we assess the direct impact of PCV13 on vaccine serotype invasive pneumococcal disease and its indirect effects on vaccine serotype invasive pneumococcal disease in populations age-ineligible for vaccination. We have leveraged our long-standing and comprehensive sentinel invasive bacterial disease surveillance, conducted after the introduction of antiretroviral therapy, to assess the direct and indirect population impact of PCV13 on the incidence of vaccine serotype and non-vaccine serotype invasive pneumococcal disease across age groups as well as individual vaccine effectiveness.

## Methods

### Study design and participants

We did a prospective observational time-series study to determine the incidence of invasive pneumococcal disease and a case-control study to estimate vaccine effectiveness in age-eligible infants (those aged 6 weeks or older on the date of PCV introduction, Nov 12, 2012).

We have conducted ongoing sentinel surveillance for laboratory-confirmed invasive pneumococcal disease, including bloodstream infection and meningitis among all age groups at the Queen Elizabeth Central Hospital (QECH), Blantyre, Malawi, since 1998, as described previously.^23^, ^24^ QECH is the government referral hospital providing free medical care to the 1·3 million urban, peri-urban, and rural residents of Blantyre District. In accordance with long-standing clinical guidelines, all adults and children presenting to QECH with fever (axillary temperature >37·5°C) or clinical evidence of sepsis or meningitis undergo blood cultures and, where appropriate, lumbar puncture. Although generally stable over time, the ratio of blood cultures to population size fell after 2006 before increasing again in 2010 and thereafter returned to a ratio similar to 2006.^23^ We report data generated after the national introduction of antiretroviral therapy, from Jan 1, 2006, to Dec 31, 2018.

The study protocol was approved by Malawi's National Health Sciences Research Committee (protocol 867), the University of Malawi College of Medicine Research and Ethics Committee (P.01/08/609 and P.09/09/826), and the University of Liverpool Research Ethics Committee (RETH490). The Centers for Disease Control and Prevention relied on the University of Liverpool Research Ethics Committee for ethics approval. For the case-control component, the parent or guardian of all child participants provided written informed consent, and children 8 years or older provided written informed assent. This consent included consent for publication.

### Procedures

Blood and cerebrospinal fluid (CSF) specimens were processed at the Malawi–Liverpool–Wellcome Trust Clinical Research Programme (co-located with QECH) laboratory, using BacT/Alert 3D (Biomerieux; Marcy l'Etoile, France). Those positive by BacT/Alert 3D were Gram stained and cocci further assessed using the catalase test. For subsequent serotyping, archived pneumococcal isolates were plated on gentamicin-sheep blood agar (SBG; 7% sheep blood, 5 μL gentamicin per mL) and incubated overnight at 37°C in 5% CO_2_. *S pneumoniae* growth was confirmed by colony morphology and optochin disc (Oxoid; Basingstoke, UK) susceptibility. The bile solubility test was used on isolates with no or intermediate (zone diameter <14mm) optochin susceptibility. A single colony of confirmed pneumococcus with the predominant morphological phenotype was selected and grown on a new SBG plate as before. Growth from this second plate was used for serotyping by latex agglutination (ImmuLex 7-10-13-valent Pneumotest; Statens Serum Institute; Copenhagen, Denmark). The ImmuLex kit allows for differential identification of each PCV13 vaccine serotype but not for differential identification of non-vaccine serotypes; therefore, non-vaccine serotype and non-typeable isolates were reported as non-vaccine serotype. Latex agglutination was used on all samples collected after Jan 1, 2014. Serotyping based on nucleic acid amplification was performed on samples collected between Jan 1, 2006, and Dec 31, 2013, using the Triplex sequential real-time PCR (rtPCR)-serotyping Africa protocol of the Centers for Disease Control and Prevention.^25^ Both assays have been shown to be highly accurate and concordant in pneumococcal serotyping.^26^, ^27^ For identified serogroups that contained vaccine serotypes but for which the rtPCR assay did not provide serotype differentiation (6A, B, C, and D, 9A and V, 18A, B, C, and F, and 7A and F), latex agglutination was used to determine vaccine serotype presence. A random selection of serotyped isolates was sent for confirmatory serotyping by Quellung reaction at the regional pneumococcal reference laboratory at the National Institute for Communicable Disease, Johannesburg, South Africa. Since Aug 13, 2011, serotyping occurs in real time with specimen processing. Isolates collected before Aug 13, 2011, were retrospectively serotyped. Demographic information was collected at the time of sampling from the patient.

### Statistical analysis

Demographic characteristics were summarised using means, SDs, medians, and IQRs for continuous variables and frequency distributions for categorical variables. Comparison of covariate distribution between study groups was done by χ^2^ test (Mantel-Haenszel χ^2^ where stratified), unless there was fewer than five observations where Fisher's exact test was used. Comparisons among continuous covariates included *t* tests of means and rank-sum tests of medians.

Invasive pneumococcal disease surveillance at QECH (providing care to the 1·3 million residents of Blantyre District, with very few inpatient beds outside QECH) included systematic recruitment from the whole population of Blantyre District. Invasive pneumococcal disease events by age category were multiplied by 100 000 and divided by annual age-specific population estimates for Blantyre District published by the Malawi National Statistics Office.^15^ Because not all historical isolates were recoverable or serotypeable, we applied each year's proportion of PCV13 vaccine serotype to impute vaccine serotype and non-vaccine serotype rates to the unserotyped pneumococcal isolates of each year by age category. The relative rarity of laboratory-confirmed invasive pneumococcal disease make incidence rates subject to annual fluctuation even in the presence of moderately large surveillance population denominators. To account for long-term trends but dampen year-on-year fluctuation we used a 3-year locally weighted moving average smoother for incidence *Î*, calculated thus:

$$\hat{I}=\frac{\left[I_{t-1}+2I_{t}+T_{t+1}\right]}{4}$$

where *I*_(t)_ indicates observed incidence at year *t*. We wexable to consistently identify people who had simultaneous meningitis and bacteraemia (ie, with both blood and CSF samples collected); thus these episodes are potentially double-counted in overall incidence calculations. Therefore, we also performed the same steps separately for pneumococci derived from blood culture and those derived from CSF (appendix p 2).

We evaluated population vaccine coverage in a convenience sample of vaccine-eligible-age children admitted with diarrhoeal disease to the same institution as invasive pneumococcal disease cases. By use of this approach we showed that community controls in Blantyre and disease-unaligned hospitalised children have comparable vaccine coverage.^28^

To estimate empirically observed invasive pneumococcal disease incidence before and after the introduction of PCV13, we fitted negative-binomial models, adjusted for time (year of sample collection), to smoothed long-term incidence of vaccine serotype and non-vaccine serotype invasive pneumococcal disease against year and vaccine introduction. To predict the counterfactual that would be expected in the period Jan 1, 2014–Dec 31, 2018, in the hypothetical absence of PCV13, we developed a model that fitted invasive pneumococcal disease incidences against year for the pre-vaccine period Jan 1, 2006–Dec 31, 2011. We compared the predicted counterfactual against the same model fitted to the smoothed empirically observed incidence for the post-PCV period, Jan 1, 2014–Dec 31, 2018, excluding the 2 years following PCV13 introduction, when vaccination coverage among infants had not yet plateaued at high population coverage. This comparison of empirically observed incidence against the counterfactual allowed us to capture not only a step change in incidence rate following vaccine, but also a change in the rate of reduction in the incidence and allowed us to observe whether vaccine introduction further enhanced the rate of reduction of an already (before PCV introduction) declining incidence. Confidence bounds about the model-derived counterfactual were constructed from the negative-binomial model. However, because we report all observed cases in the population over a period of 13 years rather than a sample thereof, we did not calculate an a-priori sample size for predefined power. Incidence rate ratios (IRRs) for invasive pneumococcal disease were calculated as:

$$\frac{\text{incidence estimated by negative binomial model}}{\text{incidence predicted by the counterfactual model}}$$

The percent reduction comparing the counterfactually predicted incidence of invasive pneumococcal disease and the empirically observed incidence of vaccine serotype invasive pneumococcal disease was calculated as (1 – IRR) × 100.

To estimate vaccine effectiveness from identified patients with invasive pneumococcal disease admitted to QECH between Nov 12, 2011 and Dec 31, 2016, we selected as study case-patients all children who had vaccine serotype invasive pneumococcal disease, who were vaccine-age-eligible (ie, born 6 weeks before PCV13 introduction or later), and whose parents consented to participate. We then conducted a random walk method in the case-patient's community to systematically identify four otherwise healthy age-matched controls, as described previously.^29^ The age difference was date of birth ±30 days among children aged younger than 1 year and ±90 days among those 1 year or older. We recorded the date of vaccination on the patient-held record (known as a Health Passport) among these controls and classified their vaccine status as it was on the admission date of the matched case. By use of STATA version 13.1, we fitted an unadjusted conditional logistic regression model comparing vaccine receipt between cases and matched controls. Vaccine effectiveness was defined as 1 minus the ratio of odds of receiving three doses of PCV13 among cases compared with matched controls. We calculated that to achieve 80% power at 5% significance to detect vaccine effectiveness of 80% against vaccine serotype invasive pneumococcal disease with four matched controls per case, vaccine uptake among controls of 60% and correlation of vaccine coverage among cases and matched controls of 0·5, we would need 16 cases of vaccine serotype invasive pneumococcal disease.

### Role of the funding source

The funder of the study had no role in study design, data collection, data analysis, data interpretation, or writing of the report.

## Results

Between Jan 1, 2006, and Dec 31, 2018, surveillance at QECH covered 10 281 476 person-years of observation. 140 498 blood cultures and 63 291 cultures of CSF were done, yielding 2638 *S pneumoniae*-positive cultures. Among these isolates, 2005 were serotyped, with 1133 (57%) being PCV13 vaccine serotype and 872 (43%) non-vaccine serotype. Serotyping results, stratified by individual serotypes (13 vaccine serotypes and non-vaccine serotype) and by age group are shown in the appendix (pp 4–18). Analysis of isolates that were and were not recoverable showed no statistically significant difference in age, gender, or sample type (data not shown). All samples sent to the South Africa regional pneumococcal reference laboratory for testing by Quellung reaction were concordant for confirmatory testing.

A reduction in total (vaccine serotype plus non-vaccine serotype) invasive pneumococcal disease incidence was observed after the national introduction of antiretroviral therapy (Jan 1, 2006) and before introduction of PCV13 (both bacteraemia and meningitis; appendix p 1). The reduction in total invasive pneumococcal disease incidence was 19% (IRR 0·81, 95% CI 0·74–0·88; p<0·0001) among infants, 14% (0·86, 0·80–0·93; p<0·0001) among children aged 1–4 years, and 8% (0·92, 0·83–1·01; p=0·084) among people aged 15 years or older. Among children aged 5–14 years there was a 2% increase in total invasive pneumococcal disease (IRR 1·02, 95% CI 0·93–1·11, p=0·72). Pre-PCV reductions in vaccine serotype and non-vaccine serotype invasive pneumococcal disease followed similar patterns (table 1). Following PCV13 introduction in November, 2011, vaccine coverage in the age-eligible birth cohort in years 2012, 2013, and 2014 for dose 1 was 84·6%, 95·1% and 97·5%, for dose 2 was 70·3%, 90·2% and 96·6%, and for dose 3 was 47·7%, 79·9% and 90·1%. In the years that followed, coverage of all doses exceeded 95%.

**Table 1:** Reductions in incidence of invasive pneumococcal disease before introduction of 13-valent pneumococcal vaccine (2006–11)

	**Incidence rate ratio (95% CI)**	**p value**
**Total**		
Infants	0·81 (0·74–0·88)	<0·0001
Children aged 1–4 years	0·86 (0·80–0·93)	<0·0001
Children aged 5–14 years	1·02 (0·93–1·11)	0·72
Individuals aged ≥15 years	0·92 (0·83–1·01)	0·084
**Vaccine serotype disease**		
Infants	0·81 (0·72–0·90)	<0·0001
Children aged 1–4 years	0·92 (0·83–1·01)	0·068
Children aged 5–14 years	1·01 (0·90–1·13)	0·89
Individuals aged ≥15 years	0·89 (0·81–0·99)	0·030
**Non-vaccine serotype disease**		
Infants	0·80 (0·75–0·86)	<0·0001
Children aged 1–4 years	0·78 (0·68–0·90)	0·0010
Children aged 5–14 years	1·04 (0·89–1·21)	0·62
Individuals aged ≥15 years	0·96 (0·85–1·08)	0·49

Data are n (%), unless otherwise specified. *χ^2^ test (Mantel-Haenszel χ^2^ where stratified) used for categorical covariates, unless fewer than five observations, where Fisher's exact test used. *t* test of means and rank-sum test of medians used for continuous covariates.

Compared with the post-vaccine, counterfactually predicted incidence of vaccine serotype invasive pneumococcal disease (ie, in hypothetical absence of vaccine), the post-vaccine empirically observed incidence of vaccine serotype disease was lower by 38% (95% CI 37–40) among infants younger than 1 year, by 74% (70–78) among children aged 1–4 years, by 79% (76–83) among children aged 5–14 years, and by 47% (44–51) among people aged 15 years or older (table 2). There was a reduction in the absolute numbers of invasive pneumococcal disease cases from 2006 to 2018 in all age groups in both vaccine serotype and non-vaccine serotype invasive pneumococcal disease. When aggregating all ages, for example, there was a reduction in confirmed cases of vaccine serotype invasive pneumococcal disease from 120 in 2006 to 13 in 2018 (appendix p 19). Similarly, confirmed cases of non-vaccine serotype invasive pneumococcal disease decreased from 102 to 14 (appendix p 19).

**Table 2:** Empirical and counterfactually predicted incidence per 100 000 age-specific population of invasive pneumococcal disease after introduction of 13-valent pneumococcal vaccine (2014–18)

	**Incidence per 100 000 age-specific population**		**Incidence rate ratio (95% CI)**
	After vaccine, empirical (95% CI)	After vaccine, counterfactual (95% CI)	
**Total**			
Infants	30·62 (30·02–31·23)	49·94 (49·34–50·54)	0·61 (0·61–0·62)
Children aged 1–4 years	7·96 (7·38–8·54)	13·74 (13·15–14·32)	0·58 (0·56–0·60)
Children aged 5–14 years	7·22 (6·57–7·88)	29·69 (29·04–30·35)	0·24 (0·23–0·26)
Individuals aged ≥15 years	10·94 (10·21–11·67)	20·29 (19·56–21·02)	0·54 (0·52–0·56)
**Vaccine serotype disease**			
Infants	15·51 (14·68–16·35)	25·18 (24·35–26·02)	0·62 (0·60–0·63)
Children aged 1–4 years	3·50 (2·80–4·21)	13·29 (12·58–14·00)	0·26 (0·22–0·30)
Children aged 5–14 years	3·76 (2·94–4·57)	18·09 (17·28–18·91)	0·21 (0·17–0·24)
Individuals aged ≥15 years	5·37 (4·61–6·13)	10·18 (9·41–10·94)	0·53 (0·49–0·56)
**Non-vaccine serotype disease**			
Infants	16·17 (15·67–16·68)	24·26 (23·75–24·76)	0·67 (0·66–0·67)
Children aged 1–4 years	4·67 (3·56–5·77)	2·50 (1·39–3·61)	1·87 (1·60–2·56)
Children aged 5–14 years	3·53 (2·44–4·63)	12·27 (11·18–13·36)	0·29 (0·22–0·35)
Individuals aged ≥15 years	5·65 (4·77–6·54)	11·19 (10·30–12·08)	0·51 (0·46–0·54)

Negative binomial regression of locally weighted incidence, adjusted for year.

Since 2015 there has been an increase in the incidence of non-vaccine serotype disease, which is now the commonest cause of invasive pneumococcal disease and led to transient increase in total invasive pneumococcal disease in 2015 and 2016. Nonetheless, total invasive pneumococcal disease incidence remained low (appendix p 1). Among 1–4-year-old and 5–14-year-old children, post-vaccine empirically observed incidence of vaccine serotype invasive pneumococcal disease was lower than was anticipated from the background (counterfactual) secular trend (figure). The model also showed a lower than anticipated incidence of non-vaccine serotype disease among 5–14-year-old children, which cannot be attributed to vaccine introduction (figure). Serotype 1 and 5 dominated in a 5-yearly cycle before vaccine introduction, with a peak still observed in serotype 1 after PCV13 introduction (appendix p 3).

**Figure:**
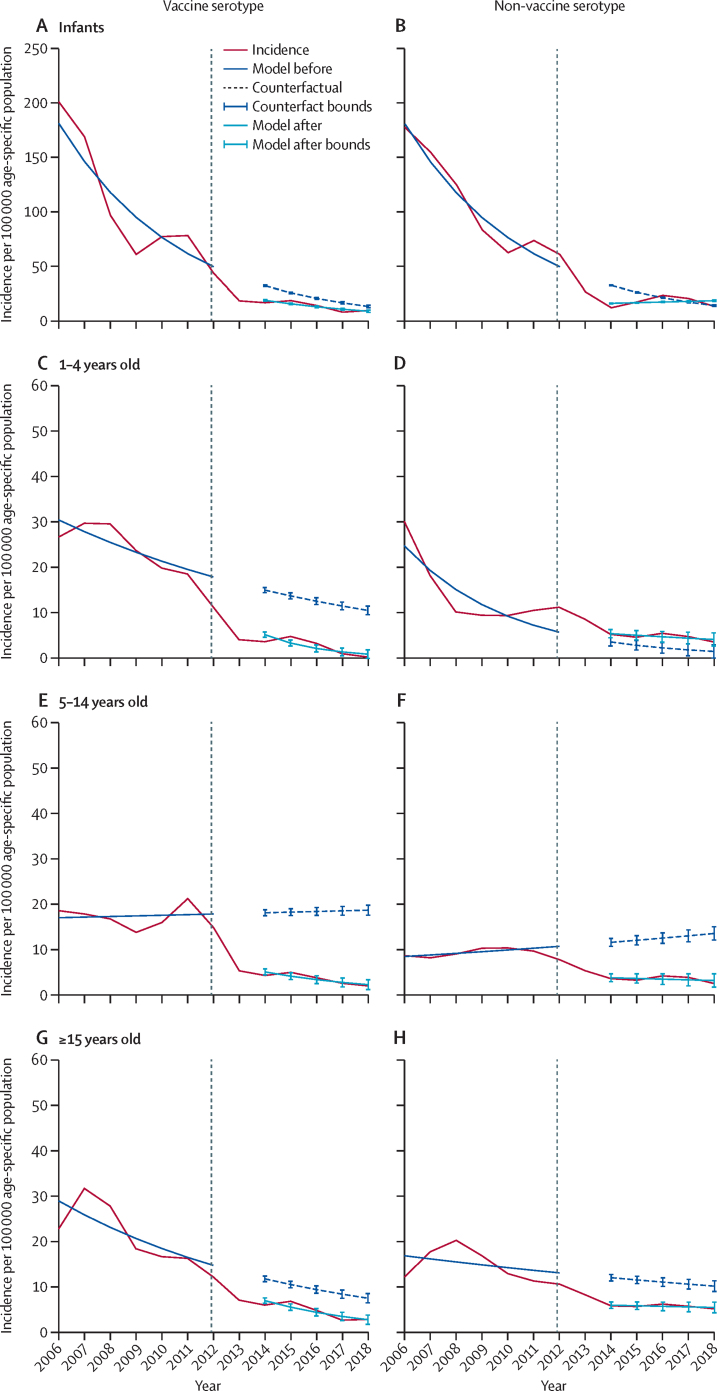
Incidence as a 3-year moving average of invasive pneumococcal disease incidence per 100 000 age-specific population from Jan 1, 2006, to Dec 31, 2018 Shown is vaccine serotype invasive pneumococcal disease in infants (A), 1–4-year-old children (C) 5–14-year-old children (E), and individuals aged 15 years and older (G) and non-vaccine serotype invasive pneumococcal disease in infants (B), 1–4-year-old children (D) 5–14-year-old children (F), and individuals aged 15 years and older (H). Negative binomial model fit for Jan 1, 2006, to Dec 31, 2011 (before PCV) and predicted counterfactual therefrom for Jan 1, 2014, to Dec 31, 2018 (after PCV). Negative binomial model fit for Jan 1, 2014, to Dec 31, 2018. PCV=pneumococcal conjugate vaccine. Vertical dashed line indicates introduction of PCV13.

Statistically significant reductions in empirically observed (non-counterfactual) incidence of vaccine serotype invasive pneumococcal disease were seen in 2014–18, starting around 2 years after introduction of PCV13 (appendix p 20). However, similar to the counterfactual analysis, reductions in empirically observed incidence were smaller and statistically non-significant among individuals younger than 1 year and those 15 years or older.

Between Oct 7, 2011, and June 27, 2016, we identified 34 cases of invasive pneumococcal disease among vaccine-eligible-age children, including 19 with vaccine serotype disease (table 3). We recruited 136 controls, matched by age and neighbourhood, between Feb 20, 2012, and May 19, 2017. Among 19 cases of vaccine serotype invasive pneumococcal disease and their 76 matched controls, 13 groups (65%) had concordant vaccination status so could not contribute statistically to conditional logistic regression. Among 37 all-serotype invasive pneumococcal disease case-control groupings, 17 (46%) had concordant vaccination statuses. Conditional logistic regression of PCV13 receipt among vaccine serotype invasive pneumococcal disease cases and their matched controls found an odds ratio of 0·19 (95% CI 0·02 to 1·74; p=0·14), equivalent to a vaccine effectiveness of 80·7% (95% CI −73·7 to 97·9). The final analysis was an unadjusted conditional logistic regression; adjusting for anthropometry, household size and mother's vital status did not materially affect the outcome (data not shown). Broken down by bacteraemia and meningitis, the observed longitudinal patterns parallel the overall results (appendix p 2)

**Table 3:** Demographic features of cases of vaccine-eligible-age all-serotype invasive pneumococcal disease and age-matched and community-matched controls

		**Cases (N=34)**	**Controls (N=136)**	p value*
Sex		..	..	0·22
	Male	23 (68%)	69 (51%)	..
	Female	11 (32%)	67 (49%)	..
Median age, months (IQR)		8·7 (5·6–18·6)	8·4 (5·3–18·9)	0·96
Vaccine record observed		33 (97%)	132 (97%)	0·065
Mean household occupants (SD)		4·3 (1·5)	4·3 (1·4)	0·99
Mean maternal age, years (SD)		25·6 (5·8)	26·5 (6·4)	0·53
Mother deceased		1 (3%)	0	0·21
Father deceased		0	1 (1%)	0·79
Z-score				
	Weight for age	−1·5	0·1	<0·0001
	Height for age	−0·3	−0·5	0·66
	Weight for height	−1·8	0·6	<0·0001
Number of pneumcoccal conjugate vaccine doses received				
	0	6 (18%)	25 (18%)	..
	1	6 (18%)	11 (8%)	..
	2	5 (15%)	15 (11%)	..
	3	17 (50%)	85 (63%)	..

Data are n (%), unless otherwise specified.

* χ^2^ test (Mantel-Haenszel χ^2^ where stratified) used for categorical covariates, unless fewer than five observations, where Fisher's exact test used. *t* test of means and rank-sum test of medians used for continuous covariates.

## Discussion

In this low-income sub-Saharan African population with a high burden of disease, in which invasive pneumococcal disease incidence was already decreasing, we used our robust long-term hospital-based surveillance to show a substantial additional reduction in the incidence of vaccine serotype invasive pneumococcal disease among children aged 1–4 years and 5–14 years following introduction of PCV13. Significant reductions in incidence did not occur among infants younger than 1 year and adolescents and adults aged 15 years or older. Although, as has been observed elsewhere,^30^ the power of our case-control analysis was undermined by high vaccine uptake (the CIs are wide and include 1), our vaccine effectiveness point estimate derived from case-control analysis suggests protection among vaccine-age-eligible children comparable with other post-introduction studies in similar populations.^5^, ^6^ Serotype replacement by non-vaccine serotype has occurred since introduction of PCV but remains low in absolute terms. The more modest indirect effects among very young and older people is similar to those seen after introduction of PCV in other sub-Saharan African countries, including The Gambia^5^ and Kenya.^6^ The reduction in the incidence of non-vaccine serotype disease in these age groups suggests a relatively small contribution of vaccine to the reduction in the incidence of vaccine serotype disease. This is in marked contrast to the USA and some European countries, where impact on adult vaccine serotype invasive pneumococcal disease exceeded that seen among vaccine-eligible age groups in absolute terms.^31^

The absence of greater evidence of indirect protection, despite high vaccine coverage in the 7 years following introduction of PCV in Malawi, might have several causes. Malawi's accelerated 3+0 vaccine schedule with no booster, and a catch-up only among infants younger than 1 year, on initial introduction has not led to the same reduction in vaccine serotype carriage seen in high-income settings where schedules frequently include doses in the second year of life.^4^ Older siblings are often the source of pneumococcal transmission to younger infants in rural Malawi. Owing to a large carriage pool in this age group, it might take many years of vaccine rollout before its effects are measurable. Additionally, median age at first pneumococcal colonisation is 6–8 weeks of age, before receipt of the full course of PCV13.^19^, ^32^ Once colonisation occurs, serotype-specific vaccine effectiveness could be blunted. Among Malawian adults, invasive pneumococcal disease is partially driven by HIV co-infection. Ubiquitous availability of antiretroviral therapy has been temporally associated with reductions in the incidence of invasive pneumococcal disease in Malawi,^14^ as has occurred in South Africa,^33^ but similar impact of antiretroviral therapy on pneumococcal carriage has not been observed.^4^, ^20^

The improved survival of HIV-infected people on antiretroviral therapy contributes to residual vaccine serotype pneumococcal carriage and transmission in the community, further reducing potential indirect vaccine effects. Adults at particularly high risk of pneumococcal colonisation and disease (including HIV-infected adults) or those whose pneumococcal acquisition is derived from other adults might benefit less from an infant schedule.^34^ Although our laboratory surveillance did not collect individual HIV status, we have reported HIV seroprevalence of 85% among adult patients with bacterial meningitis and 78% among those with pneumonia.^35^ This seroprevalence is similar to that in South Africa, where 89% of adult invasive pneumococcal disease cases were in HIV co-infected people, with invasive pneumococcal disease incidence much higher than among HIV-uninfected people.^34^ Given the persistence of vaccine serotype carriage and invasive pneumococcal disease in this vulnerable population, the possible benefit of adult vaccination in this context should be reconsidered.^13^, ^36^

Following introduction of 7-valent PCV (PCV7) in Alaska, USA, replacement disease was observed.^37^ More than a decade later, similar observations have been made in The Gambia,^38^ the UK,^12^ and South Africa^39^ following iterative introductions of PCV7 then PCV13. In Malawi, although non-vaccine serotype disease events now outnumber vaccine serotype events, the absolute number of such events is low, and overall burden of all-cause invasive pneumococcal disease has decreased. This is probably due to concurrent non-vaccine interventions, such as availability of antiretroviral therapy, rotavirus vaccination, improved control of malaria, and improved nutrition security. The cumulative impact of these public health measures in reducing the overall burden of all-cause invasive pneumococcal disease underlines that no one public health intervention (including vaccines) is adequate to significantly reduce disease burden. Rather, a strategy of implementing multiple public health interventions, including a pneumococcal vaccine that provides both direct and indirect protection, is optimal. Ongoing serotype-specific surveillance is required to monitor for the emergence of non-vaccine serotype disease, particularly for isolates that are antimicrobial resistant.

Use of observational data to attribute causality to reductions in incidence after introduction of vaccines is challenging, particularly given pre-existing reductions.^30^ Nonetheless, our surveillance has the advantages of being long standing, being methodologically stable, and occurring in a stable setting with little risk of a changes in treatment-seeking behaviour. The duration and scale of reductions in incidence of invasive pneumococcal disease before vaccine introduction is missing from many studies in Africa. Although reductions in incidence of all-serotype invasive pneumococcal disease were observed in Malawi well before introduction of PCV13, our data suggest a definitive additional benefit of vaccination. In age groups enriched for vaccine-eligible-age children where there have been high rates of vaccine uptake, invasive pneumococcal disease incidence reduced faster and was lower following vaccine introduction than anticipated from secular trends alone.

Although this work provides a robust estimate of vaccine impact, it has several limitations. We were not able to correct for episodes of concurrent bacteraemia and meningitis, but we suspect this number is small and consistent over time and unlikely to introduce substantial bias. A vaccine with high uptake and even moderate impact within a population can undermine the ability to assess its effectiveness in individual recipients using a case-control design. The estimated vaccine uptake of 60% among controls for the purpose of sample size calculation was too conservative, given Malawi's successful rapid PCV13 roll out and high coverage. This smaller sample size challenged the analytical power of the matched case-control study which requires discordance in coverage. A successful vaccine decreases risk of disease, which, together with the long-standing gradual reductions in invasive pneumococcal disease incidence, made it difficult to recruit sufficient vaccine-eligible-age cases of vaccine serotype invasive pneumococcal disease for the case-control study.^30^ The resulting insufficient recruitment of cases led to the case-control study being underpowered. In populations with high vaccine coverage, there is always a concern about the comparability of unvaccinated individuals with those who are vaccinated. Notwithstanding these limitations, taken together the observed impact and effectiveness are consistent in suggesting substantial reductions in risk of vaccine serotype invasive pneumococcal disease in Malawi among vaccine-eligible age groups.

In conclusion, even with pre-existing reductions in the incidence of invasive pneumococcal disease, the introduction of a 3+0 PCV13 infant schedule in Malawi has led to a substantial reduction in the incidence of invasive pneumococcal disease among vaccine-eligible-age children. However, despite high vaccination coverage and adequate time since PCV introduction, indirect protection among adolescents and adults has been more muted, especially in contrast with that seen in high-income countries. A trend of decreasing invasive pneumococcal disease incidence before introduction of PCV further underlines the need for a strategy of implementing multiple public health interventions to substantially reduce disease burden. Nonetheless, as improvements in available vaccine interventions arise, including alternative schedules and extended-spectrum vaccines at reduced costs, consideration should be given to evaluating PCV vaccination among high-risk populations, including HIV-infected adults, in settings with high force of infection and disease burden.

## Data sharing

The data supporting the findings of this study have been deposited in the supplementary material and in a Figshare data repository.

## Declaration of interests

NF has received investigator-initiated research grants from GlaxoSmithKline Biologicals, outside of the submitted work. NAC reports receiving investigator-initiated grants and non-financial support from GlaxoSmithKline Biologicals, outside the submitted work. NB-Z reports investigator-initiated research grants from Merck and Serum Institute of India, outside the submitted work. AvG has received research grants from Pfizer and Sanofi, outside the submitted work. All other authors declare no competing interests.
