# Correction to *Lancet Glob Health* 2021; 9: e989–98

**DOI:** 10.1016/S2214-109X(21)00469-1

**Published:** 2021-11-16

*Bar-Zeev N, Swarthout TD, Everett DB, et al. Impact and effectiveness of 13-valent pneumococcal conjugate vaccine on population incidence of vaccine and non-vaccine serotype invasive pneumococcal disease in Blantyre, Malawi, 2006–18: prospective observational time-series and case-control studies.* Lancet Glob Health *2021; **9:** e989–98—*An incorrect version of this Article's appendix was uploaded. The appendix has been corrected as of Nov 16, 2021.

