# Supplementary material for: Impact and effectiveness of 13-valent pneumococcal conjugate vaccine on population incidence of vaccine and non-vaccine serotype invasive pneumococcal disease in Blantyre, Malawi, 2006–18: prospective observational time-series and case-control studies
Source: Lancet Glob Health. 2021 Jun 15;9(7):e989–98. doi: 10.1016/S2214-109X(21)00165-0 (PMC8220129; doi:10.1016/S2214-109X(21)00165-0)
Supplement: Supplementary appendix [file mmc1.pdf]

THE LANCET
Global Health

Supplementary appendix

This appendix formed part of the original submission and has been peer reviewed. We post it as supplied by the authors.

Supplement to: Bar-Zeev N, Swarthout TD, Everett DB, et al. Impact and effectiveness of 13-valent pneumococcal conjugate vaccine on population incidence of vaccine and non-vaccine serotype invasive pneumococcal disease in Blantyre, Malawi, 2006–18: prospective observational time-series and case-control studies. *Lancet Glob Health* 2021; **9:** e989–98.

Supplementary Material
Bar-Zeev N, Swarthout TD, et al.

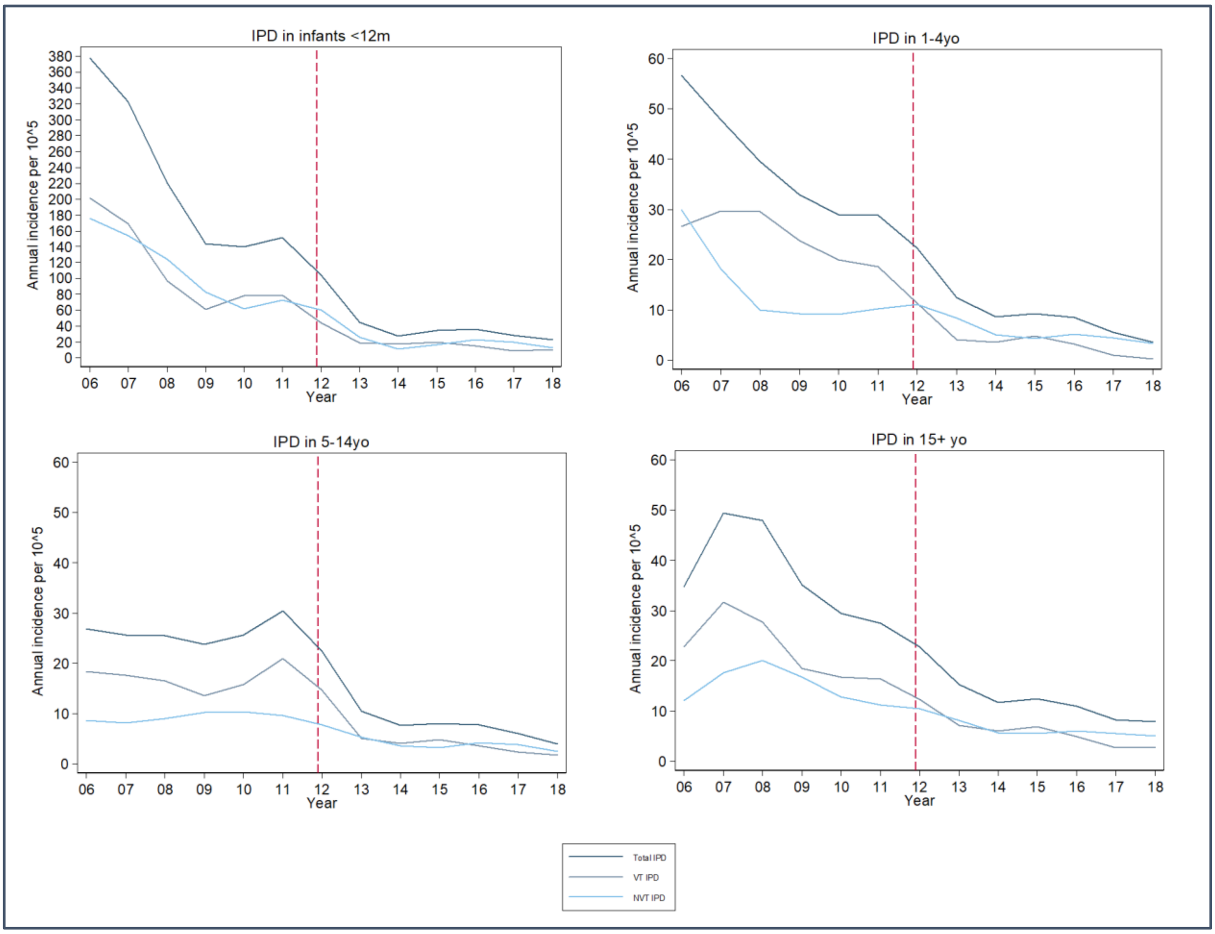

Supplementary Figure 1. Three-year moving average smoothed incidence per 100,000 age-specific population of invasive pneumococcal disease (IPD) in Blantyre, Malawi, 1 January 2006 to 31 December 2018. Dashed red line indicates introduction of 13-valent Pneumococcal Conjugate Vaccine. Note: Y-axis in infant panel differs to other panels. IPD = invasive pneumococcal disease; VT = 13-valent vaccine serotypes; NVT = non-13-valent vaccine serotypes.

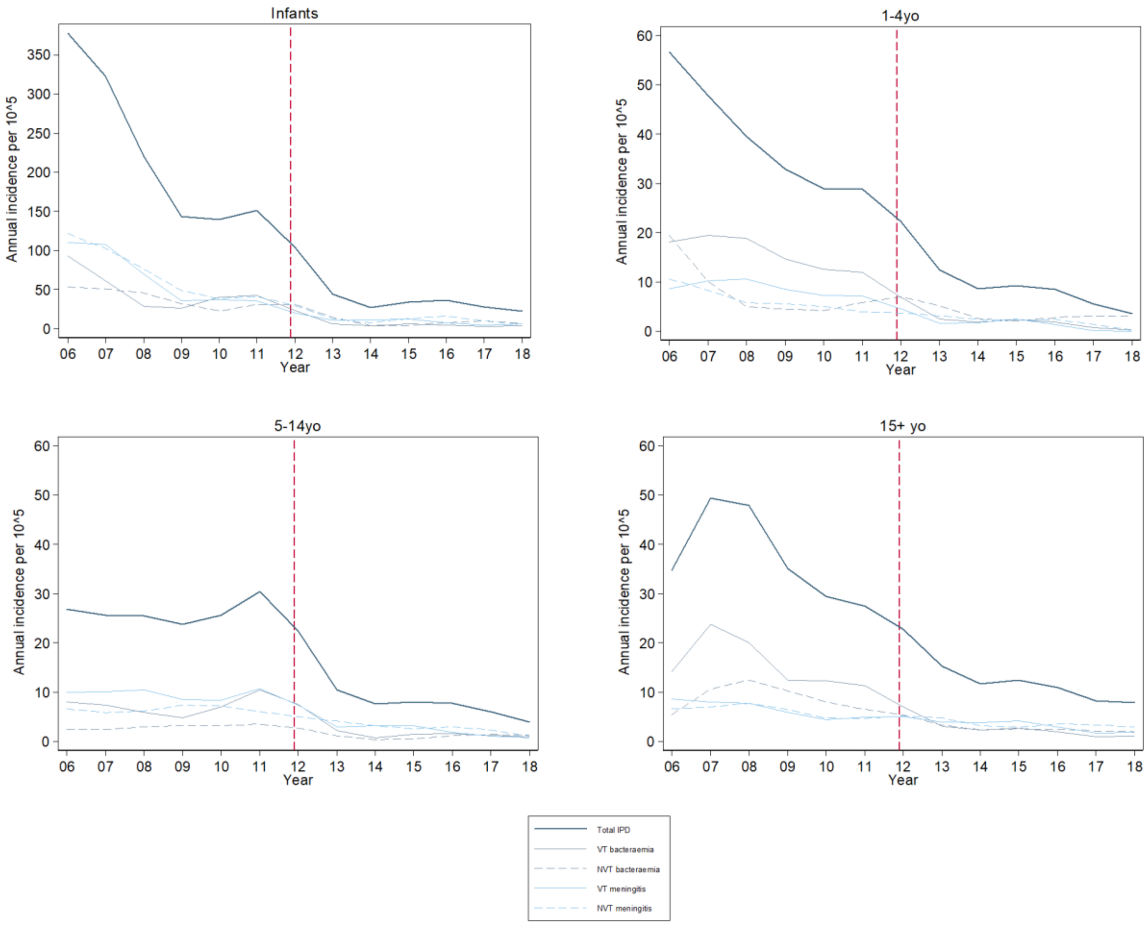

Supplementary Figure 2. Three-year moving average smoothed incidence per 100,000 age-specific population of pneumococcal bacteraemia and meningitis, Blantyre, Malawi, January 1 2006 to December 31 2018. Dashed red line indicates introduction of 13-valent Pneumococcal Conjugate Vaccine. IPD = invasive pneumococcal disease; VT = 13-valent vaccine serotypes; NVT = non-13-valent vaccine serotypes.

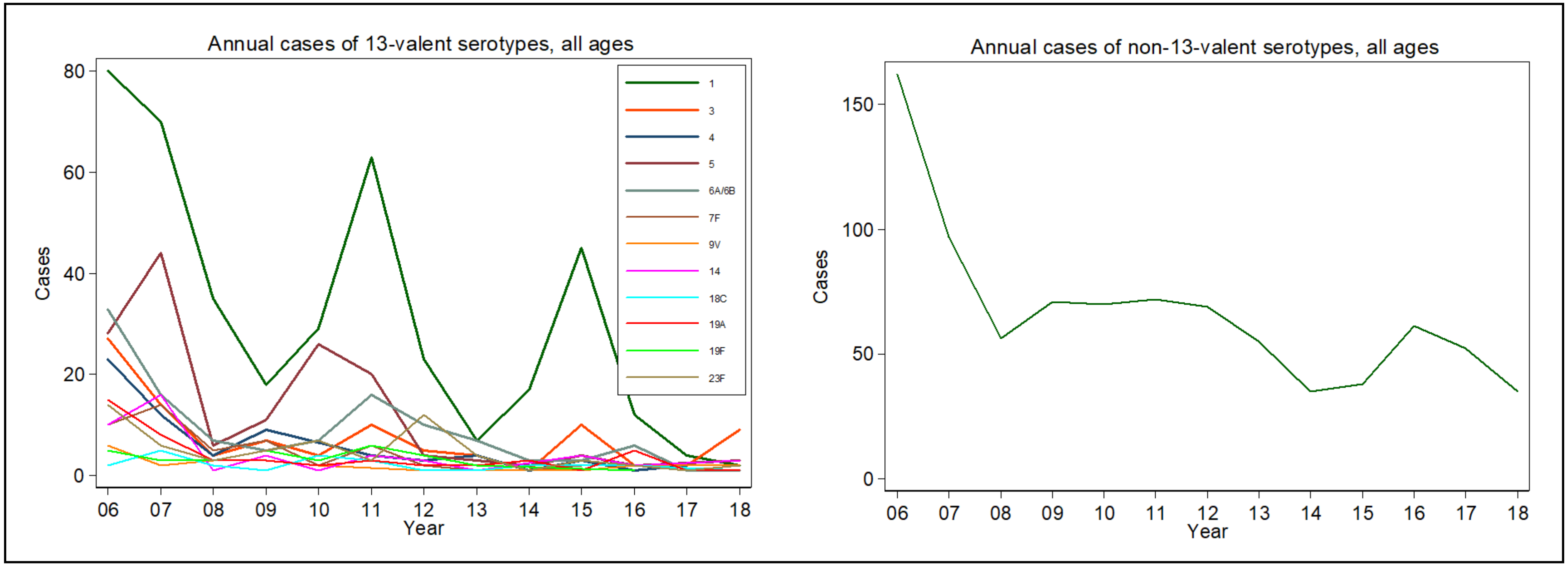

Supplementary Figure 3. Serotype specific cases by year, Blantyre, Malawi, January 1 2006 to December 31 2018.

Serotyping results, All ages

Supplementary Table 1a. Frequency of serotypes stratified by year

VT	2006	2007	2008	2009	2010	2011	2012	2013	2014	2015	2016	2017	2018	Total
1	80	70	35	18	29	63	23	7	17	45	12	4	2	405
3	27	14	4	7	4	10	5	4	1	10	2	2	9	99
4	23	12	4	9	0	4	3	4	1	3	1	2	0	66
5	28	44	6	11	26	20	4	3	2	4	2	0	3	153
6A	15	5	3	1	2	7	6	3	2	1	4	1	1	51
6A/6B	0	0	0	2	2	1	0	1	0	0	0	0	0	6
6B	18	11	4	2	3	8	4	3	1	2	2	0	0	58
7F	10	14	5	7	2	6	2	1	1	2	2	2	2	56
9V	6	2	3	3	2	0	1	1	1	1	2	2	2	26
14	10	16	1	4	1	4	3	1	0	4	2	0	3	49
18C	2	5	2	1	4	3	1	1	2	0	2	0	1	24
19A	15	8	3	3	2	3	2	2	3	1	5	1	1	49
19F	5	3	3	5	3	6	0	2	0	0	1	0	0	28
23F	14	6	3	5	7	3	12	4	1	3	2	1	2	63
Total VT	253	210	76	78	87	138	66	37	32	76	39	15	26	1133
NVT	162	97	56	71	70	72	69	55	35	38	60	52	35	872
Recovered	415	307	132	149	157	210	135	92	67	114	99	67	61	2005
Unrecovered	49	52	117	47	17	15	15	5	5	10	3	2	5	342

Supplementary Table 1b. Prevalence of VT as portion of VT carriage

VT	2006	2007	2008	2009	2010	2011	2012	2013	2014	2015	2016	2017	2018	Total
1	31.6	33.3	46.1	23.1	33.3	45.7	34.9	18.9	53.1	59.2	30.8	26.7	7.7	35.8
3	10.7	6.7	5.3	9.0	4.6	7.3	7.6	10.8	3.1	13.2	5.1	13.3	34.6	8.7
4	9.1	5.7	5.3	11.5	0.0	2.9	4.6	10.8	3.1	4.0	2.6	13.3	0.0	5.8
5	11.1	21.0	7.9	14.1	29.9	14.5	6.1	8.1	6.3	5.3	5.1	0.0	11.5	13.5
6A	5.9	2.4	4.0	1.3	2.3	5.1	9.1	8.1	6.3	1.3	10.3	6.7	3.9	4.5
6A/6B	0.0	0.0	0.0	2.6	2.3	0.7	0.0	2.7	0.0	0.0	0.0	0.0	0.0	0.5
6B	7.1	5.2	5.3	2.6	3.5	5.8	6.1	8.1	3.1	2.6	5.1	0.0	0.0	5.1
7F	4.0	6.7	6.6	9.0	2.3	4.4	3.0	2.7	3.1	2.6	5.1	13.3	7.7	4.9
9V	2.4	1.0	4.0	3.9	2.3	0.0	1.5	2.7	3.1	1.3	5.1	13.3	7.7	2.3
14	4.0	7.6	1.3	5.1	1.2	2.9	4.6	2.7	0.0	5.3	5.1	0.0	11.5	4.3
18C	0.8	2.4	2.6	1.3	4.6	2.2	1.5	2.7	6.3	0.0	5.1	0.0	3.9	2.1
19A	5.9	3.8	4.0	3.9	2.3	2.2	3.0	5.4	9.4	1.3	12.8	6.7	3.9	4.3
19F	2.0	1.4	4.0	6.4	3.5	4.4	0.0	5.4	0.0	0.0	2.6	0.0	0.0	2.5
23F	5.5	2.9	4.0	6.4	8.1	2.2	18.2	10.8	3.1	4.0	5.1	6.7	7.7	5.6
Total	100	100	100	100	100	100	100	100	100	100	100	100	100	100

Supplementary Table 1c. Prevalence of VT as portion of total carriage

VT	2006	2007	2008	2009	2010	2011	2012	2013	2014	2015	2016	2017	2018	Total
1	19.3	22.8	26.5	12.1	18.5	30.0	17.0	7.6	25.4	39.5	12.1	6.0	3.3	20.2
3	6.5	4.6	3.0	4.7	2.6	4.8	3.7	4.4	1.5	8.8	2.0	3.0	14.8	4.9
4	5.5	3.9	3.0	6.0	0.0	1.9	2.2	4.4	1.5	2.6	1.0	3.0	0.0	3.3
5	6.8	14.3	4.6	7.4	16.6	9.5	3.0	3.3	3.0	3.5	2.0	0.0	4.9	7.6
6A	3.6	1.6	2.3	0.7	1.3	3.3	4.4	3.3	3.0	0.9	4.0	1.5	1.6	2.5
6A/6B	0.0	0.0	0.0	1.3	1.3	0.5	0.0	1.1	0.0	0.0	0.0	0.0	0.0	0.3
6B	4.3	3.6	3.0	1.3	1.9	3.8	3.0	3.3	1.5	1.8	2.0	0.0	0.0	2.9
7F	2.4	4.6	3.8	4.7	1.3	2.9	1.5	1.1	1.5	1.8	2.0	3.0	3.3	2.8
9V	1.5	0.7	2.3	2.0	1.3	0.0	0.7	1.1	1.5	0.9	2.0	3.0	3.3	1.3
14	2.4	5.2	0.8	2.7	0.6	1.9	2.2	1.1	0.0	3.5	2.0	0.0	4.9	2.4
18C	0.5	1.6	1.5	0.7	2.6	1.4	0.7	1.1	3.0	0.0	2.0	0.0	1.6	1.2
19A	3.6	2.6	2.3	2.0	1.3	1.4	1.5	2.2	4.5	0.9	5.1	1.5	1.6	2.4
19F	1.2	1.0	2.3	3.4	1.9	2.9	0.0	2.2	0.0	0.0	1.0	0.0	0.0	1.4
23F	3.4	2.0	2.3	3.4	4.5	1.4	8.9	4.4	1.5	2.6	2.0	1.5	3.3	3.1
Total VT	61.0	68.4	57.6	52.3	55.4	65.7	48.9	40.2	47.8	66.7	39.4	22.4	42.6	56.5

Supplementary Table 1d. Prevalence of NVT as portion of total carriage

	2006	2007	2008	2009	2010	2011	2012	2013	2014	2015	2016	2017	2018	Total
NVT	39.0	31.6	42.5	47.6	44.6	34.3	51.1	59.8	52.2	33.3	60.6	77.6	57.4	43.5

Infants <1yr old

Supplementary Table 2a. Frequency of serotypes, stratified by year

VT	2006	2007	2008	2009	2010	2011	2012	2013	2014	2015	2016	2017	2018	Total
1	2	2	0	0	1	1	0	0	1	2	0	0	0	9
3	2	0	1	0	0	1	0	0	0	3	0	0	0	7
4	0	1	0	0	0	2	0	0	0	0	0	0	0	3
5	7	13	0	3	4	7	1	0	1	0	1	0	2	39
6A	2	1	0	0	0	1	0	0	1	0	0	0	0	5
6B	3	1	1	0	0	2	1	2	0	0	1	0	0	11
7F	2	1	0	1	0	1	0	0	0	0	0	0	0	5
9V	0	0	0	0	0	0	0	0	0	0	0	0	0	0
14	0	3	1	0	0	1	0	1	0	0	1	0	1	8
18C	1	0	1	0	0	1	0	0	1	0	1	0	0	5
19A	1	2	0	0	0	0	0	0	0	0	0	0	0	3
19F	2	1	0	0	0	1	0	0	0	0	1	0	0	5
23F	2	1	0	0	4	0	3	0	0	0	0	0	1	11
Total VT	24	26	4	4	9	18	5	3	4	5	5	0	4	111
NVT	23	18	8	6	4	17	10	4	1	5	5	8	2	111
Recovered	47	44	12	10	13	35	15	7	5	10	10	8	6	222
Unrecovered	13	8	12	0	0	2	5	2	1	0	2	0	1	46

Supplementary Table 2b. Prevalence of VT as portion of VT carriage, stratified by year

VT	2006	2007	2008	2009	2010	2011	2012	2013	2014	2015	2016	2017	2018	Total
1	8.3	7.7	0.0	0.0	11.1	5.6	0.0	0.0	25.0	40.0	0.0	0.0	0.0	8.1
3	8.3	0.0	25.0	0.0	0.0	5.6	0.0	0.0	0.0	60.0	0.0	0.0	0.0	6.3
4	0.0	3.8	0.0	0.0	0.0	11.1	0.0	0.0	0.0	0.0	0.0	0.0	0.0	2.7
5	29.2	50.0	0.0	75.0	44.4	38.9	20.0	0.0	25.0	0.0	20.0	0.0	50.0	35.1
6A	8.3	3.8	0.0	0.0	0.0	5.6	0.0	0.0	25.0	0.0	0.0	0.0	0.0	4.5
6B	12.5	3.8	25.0	0.0	0.0	11.1	20.0	66.7	0.0	0.0	20.0	0.0	0.0	9.9
7F	8.3	3.8	0.0	25.0	0.0	5.6	0.0	0.0	0.0	0.0	0.0	0.0	0.0	4.5
9V	0.0	0.0	0.0	0.0	0.0	0.0	0.0	0.0	0.0	0.0	0.0	0.0	0.0	0.0
14	0.0	11.5	25.0	0.0	0.0	5.6	0.0	33.3	0.0	0.0	20.0	0.0	25.0	7.2
18C	4.2	0.0	25.0	0.0	0.0	5.6	0.0	0.0	25.0	0.0	20.0	0.0	0.0	4.5
19A	4.2	7.7	0.0	0.0	0.0	0.0	0.0	0.0	0.0	0.0	0.0	0.0	0.0	2.7
19F	8.3	3.8	0.0	0.0	0.0	5.6	0.0	0.0	0.0	0.0	20.0	0.0	0.0	4.5
23F	8.3	3.8	0.0	0.0	44.4	0.0	60.0	0.0	0.0	0.0	0.0	0.0	25.0	9.9
Total VT	100	100	100	100	100	100	100	100	100	100	100	0	100	100

Supplementary Table 2c. Prevalence of VT as portion of total carriage, stratified by year

VT	2006	2007	2008	2009	2010	2011	2012	2013	2014	2015	2016	2017	2018	Total
1	4.3	4.5	0.0	0.0	7.7	2.9	0.0	0.0	20.0	20.0	0.0	0.0	0.0	4.1
3	4.3	0.0	8.3	0.0	0.0	2.9	0.0	0.0	0.0	30.0	0.0	0.0	0.0	3.2
4	0.0	2.3	0.0	0.0	0.0	5.7	0.0	0.0	0.0	0.0	0.0	0.0	0.0	1.4
5	14.9	29.5	0.0	30.0	30.8	20.0	6.7	0.0	20.0	0.0	10.0	0.0	33.3	17.6
6A	4.3	2.3	0.0	0.0	0.0	2.9	0.0	0.0	20.0	0.0	0.0	0.0	0.0	2.3
6B	6.4	2.3	8.3	0.0	0.0	5.7	6.7	28.6	0.0	0.0	10.0	0.0	0.0	5.0
7F	4.3	2.3	0.0	10.0	0.0	2.9	0.0	0.0	0.0	0.0	0.0	0.0	0.0	2.3
9V	0.0	0.0	0.0	0.0	0.0	0.0	0.0	0.0	0.0	0.0	0.0	0.0	0.0	0.0
14	0.0	6.8	8.3	0.0	0.0	2.9	0.0	14.3	0.0	0.0	10.0	0.0	16.7	3.6
18C	2.1	0.0	8.3	0.0	0.0	2.9	0.0	0.0	20.0	0.0	10.0	0.0	0.0	2.3
19A	2.1	4.5	0.0	0.0	0.0	0.0	0.0	0.0	0.0	0.0	0.0	0.0	0.0	1.4
19F	4.3	2.3	0.0	0.0	0.0	2.9	0.0	0.0	0.0	0.0	10.0	0.0	0.0	2.3
23F	4.3	2.3	0.0	0.0	30.8	0.0	20.0	0.0	0.0	0.0	0.0	0.0	16.7	5.0
Total VT	51.1	59.1	33.3	40.0	69.2	51.4	33.3	42.9	80.0	50.0	50.0	0.0	66.7	50.0

Supplementary Table 2d. Prevalence of NVT as portion of total carriage, stratified by year

	2006	2007	2008	2009	2010	2011	2012	2013	2014	2015	2016	2017	2018	Total
NVT	48.9	40.9	66.7	60.0	30.8	48.6	66.7	57.1	20.0	50.0	50.0	100.0	33.3	100.0

Aged 1-4 years old

Supplementary Table 3a. Frequency of serotypes, stratified by year

VT	2006	2007	2008	2009	2010	2011	2012	2013	2014	2015	2016	2017	2018	Total
1	1	4	2	2	1	8	1	1	0	4	0	0	0	24
3	1	0	0	0	0	0	0	0	0	1	0	0	0	2
4	0	0	0	0	0	0	0	0	0	0	0	0	0	0
5	1	4	2	1	2	1	0	1	1	1	0	0	0	14
6A	0	0	1	0	0	2	2	1	0	0	1	0	0	7
6A/6B	0	0	0	1	0	1	0	0	0	0	0	0	0	2
6B	3	0	0	0	2	1	0	0	0	0	0	0	0	6
7F	1	0	0	1	0	1	0	0	0	0	0	0	0	3
9V	1	0	0	0	0	0	0	0	0	1	1	0	0	3
14	0	2	0	1	0	0	2	0	0	0	0	0	0	5
18C	0	1	0	0	1	1	0	0	0	0	0	0	0	3
19A	0	1	0	0	0	0	0	0	0	0	0	0	0	1
19F	0	1	0	2	0	1	0	0	0	0	0	0	0	4
23F	1	1	1	3	1	0	1	1	1	1	1	1	0	13
Total	9	14	6	11	7	16	6	4	2	8	3	1	0	87
NVT	15	5	2	4	5	5	11	8	5	3	8	4	4	79
Recovered	24	19	8	15	12	21	17	12	7	11	11	5	4	166
Unrecovered	6	5	12	4	3	1	2	0	1	1	1	1	0	37

Supplementary Table 3b. Prevalence of VT as portion of VT carriage, stratified by year

Serotype	2006	2007	2008	2009	2010	2011	2012	2013	2014	2015	2016	2017	2018	Total
1	11.1	28.6	33.3	18.2	14.3	50.0	16.7	25.0	0.0	50.0	0.0	0.0	0.0	27.6
3	11.1	0.0	0.0	0.0	0.0	0.0	0.0	0.0	0.0	12.5	0.0	0.0	0.0	2.3
4	0.0	0.0	0.0	0.0	0.0	0.0	0.0	0.0	0.0	0.0	0.0	0.0	0.0	0.0
5	11.1	28.6	33.3	9.1	28.6	6.3	0.0	25.0	50.0	12.5	0.0	0.0	0.0	16.1
6A	0.0	0.0	16.7	0.0	0.0	12.5	33.3	25.0	0.0	0.0	33.3	0.0	0.0	8.0
6A/6B	0.0	0.0	0.0	9.1	0.0	6.3	0.0	0.0	0.0	0.0	0.0	0.0	0.0	2.3
6B	33.3	0.0	0.0	0.0	28.6	6.3	0.0	0.0	0.0	0.0	0.0	0.0	0.0	6.9
7F	11.1	0.0	0.0	9.1	0.0	6.3	0.0	0.0	0.0	0.0	0.0	0.0	0.0	3.4
9V	11.1	0.0	0.0	0.0	0.0	0.0	0.0	0.0	0.0	12.5	33.3	0.0	0.0	3.4
14	0.0	14.3	0.0	9.1	0.0	0.0	33.3	0.0	0.0	0.0	0.0	0.0	0.0	5.7
18C	0.0	7.1	0.0	0.0	14.3	6.3	0.0	0.0	0.0	0.0	0.0	0.0	0.0	3.4
19A	0.0	7.1	0.0	0.0	0.0	0.0	0.0	0.0	0.0	0.0	0.0	0.0	0.0	1.1
19F	0.0	7.1	0.0	18.2	0.0	6.3	0.0	0.0	0.0	0.0	0.0	0.0	0.0	4.6
23F	11.1	7.1	16.7	27.3	14.3	0.0	16.7	25.0	50.0	12.5	33.3	100.0	0.0	14.9
Total	100	100	100	100	100	100	100	100	100	100	100	100	0	100

Supplementary Table 3c. Prevalence of VT as portion of total carriage, stratified by year

Serotype	2006	2007	2008	2009	2010	2011	2012	2013	2014	2015	2016	2017	2018	Total
1	4.2	21.1	25.0	13.3	8.3	38.1	5.9	8.3	0.0	36.4	0.0	0.0	0.0	14.5
3	4.2	0.0	0.0	0.0	0.0	0.0	0.0	0.0	0.0	9.1	0.0	0.0	0.0	1.2
4	0.0	0.0	0.0	0.0	0.0	0.0	0.0	0.0	0.0	0.0	0.0	0.0	0.0	0.0
5	4.2	21.1	25.0	6.7	16.7	4.8	0.0	8.3	14.3	9.1	0.0	0.0	0.0	8.4
6A	0.0	0.0	12.5	0.0	0.0	9.5	11.8	8.3	0.0	0.0	9.1	0.0	0.0	4.2
6A/6B	0.0	0.0	0.0	6.7	0.0	4.8	0.0	0.0	0.0	0.0	0.0	0.0	0.0	1.2
6B	12.5	0.0	0.0	0.0	16.7	4.8	0.0	0.0	0.0	0.0	0.0	0.0	0.0	3.6
7F	4.2	0.0	0.0	6.7	0.0	4.8	0.0	0.0	0.0	0.0	0.0	0.0	0.0	1.8
9V	4.2	0.0	0.0	0.0	0.0	0.0	0.0	0.0	0.0	9.1	9.1	0.0	0.0	1.8
14	0.0	10.5	0.0	6.7	0.0	0.0	11.8	0.0	0.0	0.0	0.0	0.0	0.0	3.0
18C	0.0	5.3	0.0	0.0	8.3	4.8	0.0	0.0	0.0	0.0	0.0	0.0	0.0	1.8
19A	0.0	5.3	0.0	0.0	0.0	0.0	0.0	0.0	0.0	0.0	0.0	0.0	0.0	0.6
19F	0.0	5.3	0.0	13.3	0.0	4.8	0.0	0.0	0.0	0.0	0.0	0.0	0.0	2.4
23F	4.2	5.3	12.5	20.0	8.3	0.0	5.9	8.3	14.3	9.1	9.1	20.0	0.0	7.8
Total	37.5	73.7	75.0	73.3	58.3	76.2	35.3	33.3	28.6	72.7	27.3	20.0	0.0	52.4

Supplementary Table 3d. Prevalence of NVT as portion of total carriage, stratified by year

	2006	2007	2008	2009	2010	2011	2012	2013	2014	2015	2016	2017	2018	Total
NVT	62.5	26.3	25.0	26.7	41.7	23.8	64.7	66.7	71.4	27.3	72.7	80.0	100.0	47.6

Aged 5-14 years old

Supplementary Table 4a. Frequency of serotypes, stratified by year

VT	2006	2007	2008	2009	2010	2011	2012	2013	2014	2015	2016	2017	2018	Total
1	8	7	4	2	7	21	4	0	6	8	4	1	2	74
3	0.0	0.0	0.0	1	0.0	3	0.0	0.0	0.0	0.0	0.0	0.0	0.0	4
4	3	0.0	0.0	2	0.0	0.0	2	0.0	1	1	0.0	2	0.0	11
5	0.0	2	0.0	0.0	4	2	2	1	0.0	0.0	0.0	0.0	0.0	11
6A	3	0.0	0.0	0.0	0.0	1	2	0.0	0.0	0.0	0.0	0.0	0.0	6
6B	0.0	1	0.0	0.0	0.0	2	1	0.0	1	0.0	0.0	0.0	0.0	5
7F	0.0	2	1	1	0.0	1	1	0.0	1	0.0	2	2	0.0	11
9V	0.0	0.0	0.0	0.0	1	0.0	1	0.0	1	0.0	1	0.0	0.0	4
14	0.0	2	0.0	1	0.0	1	0.0	0.0	0.0	1	0.0	0.0	0.0	5
18C	0.0	0.0	1	0.0	1	0.0	1	0.0	0.0	0.0	0.0	0.0	1	4
19A	2	0.0	0.0	0.0	0.0	1	1	0.0	0.0	0.0	1	0.0	0.0	5
19F	0.0	0.0	3	3	1	0.0	0.0	0.0	0.0	0.0	0.0	0.0	0.0	7
23F	2	0.0	1	0.0	0.0	3	3	0.0	0.0	0.0	0.0	0.0	0.0	9
Total	18	14	10	10	14	35	18	1	10	10	8	5	3	156
NVT	9	6	5	9	13	12	10	9	8	4	11	10	3	109
Recovered	27	20	15	19	27	47	28	10	18	14	19	15	6	265
Unrecovered	5	5	21	7	1	4	4	0.0	0.0	1	0.0	0.0	1	49

Supplementary Table 4b. Prevalence of VT as portion of VT carriage, stratified by year

Serotype	2006	2007	2008	2009	2010	2011	2012	2013	2014	2015	2016	2017	2018	Total
1	44.4	50.0	40.0	20.0	50.0	60.0	22.2	0.0	60.0	80.0	50.0	20.0	66.7	47.4
3	0.0	0.0	0.0	10.0	0.0	8.6	0.0	0.0	0.0	0.0	0.0	0.0	0.0	2.6
4	16.7	0.0	0.0	20.0	0.0	0.0	11.1	0.0	10.0	10.0	0.0	40.0	0.0	7.1
5	0.0	14.3	0.0	0.0	28.6	5.7	11.1	100.0	0.0	0.0	0.0	0.0	0.0	7.1
6A	16.7	0.0	0.0	0.0	0.0	2.9	11.1	0.0	0.0	0.0	0.0	0.0	0.0	3.8
6B	0.0	7.1	0.0	0.0	0.0	5.7	5.6	0.0	10.0	0.0	0.0	0.0	0.0	3.2
7F	0.0	14.3	10.0	10.0	0.0	2.9	5.6	0.0	10.0	0.0	25.0	40.0	0.0	7.1
9V	0.0	0.0	0.0	0.0	7.1	0.0	5.6	0.0	10.0	0.0	12.5	0.0	0.0	2.6
14	0.0	14.3	0.0	10.0	0.0	2.9	0.0	0.0	0.0	10.0	0.0	0.0	0.0	3.2
18C	0.0	0.0	10.0	0.0	7.1	0.0	5.6	0.0	0.0	0.0	0.0	0.0	33.3	2.6
19A	11.1	0.0	0.0	0.0	0.0	2.9	5.6	0.0	0.0	0.0	12.5	0.0	0.0	3.2
19F	0.0	0.0	30.0	30.0	7.1	0.0	0.0	0.0	0.0	0.0	0.0	0.0	0.0	4.5
23F	11.1	0.0	10.0	0.0	0.0	8.6	16.7	0.0	0.0	0.0	0.0	0.0	0.0	5.8
**Total**	**100**	**100**	**100**	**100**	**100**	**100**	**100**	**100**	**100**	**100**	**100**	**100**	**100**	**100**

Supplementary Table 4c. Prevalence of VT as portion of total carriage, stratified by year

Serotype	2006	2007	2008	2009	2010	2011	2012	2013	2014	2015	2016	2017	2018	Total
1	29.6	35.0	26.7	10.5	25.9	44.7	14.3	0.0	33.3	57.1	21.1	6.7	33.3	27.9
3	0.0	0.0	0.0	5.3	0.0	6.4	0.0	0.0	0.0	0.0	0.0	0.0	0.0	1.5
4	11.1	0.0	0.0	10.5	0.0	0.0	7.1	0.0	5.6	7.1	0.0	13.3	0.0	4.2
5	0.0	10.0	0.0	0.0	14.8	4.3	7.1	10.0	0.0	0.0	0.0	0.0	0.0	4.2
6A	11.1	0.0	0.0	0.0	0.0	2.1	7.1	0.0	0.0	0.0	0.0	0.0	0.0	2.3
6B	0.0	5.0	0.0	0.0	0.0	4.3	3.6	0.0	5.6	0.0	0.0	0.0	0.0	1.9
7F	0.0	10.0	6.7	5.3	0.0	2.1	3.6	0.0	5.6	0.0	10.5	13.3	0.0	4.2
9V	0.0	0.0	0.0	0.0	3.7	0.0	3.6	0.0	5.6	0.0	5.3	0.0	0.0	1.5
14	0.0	10.0	0.0	5.3	0.0	2.1	0.0	0.0	0.0	7.1	0.0	0.0	0.0	1.9
18C	0.0	0.0	6.7	0.0	3.7	0.0	3.6	0.0	0.0	0.0	0.0	0.0	16.7	1.5
19A	7.4	0.0	0.0	0.0	0.0	2.1	3.6	0.0	0.0	0.0	5.3	0.0	0.0	1.9
19F	0.0	0.0	20.0	15.8	3.7	0.0	0.0	0.0	0.0	0.0	0.0	0.0	0.0	2.6
23F	7.4	0.0	6.7	0.0	0.0	6.4	10.7	0.0	0.0	0.0	0.0	0.0	0.0	3.4
Total	66.7	70.0	66.7	52.6	51.9	74.5	64.3	10.0	55.6	71.4	42.1	33.3	50.0	58.9

Supplementary Table 4d. Prevalence of NVT as portion of total carriage, stratified by year

	2006	2007	2008	2009	2010	2011	2012	2013	2014	2015	2016	2017	2018	Total
NVT	33.3	30.0	33.3	47.4	48.1	25.5	35.7	90.0	44.4	28.6	57.9	66.7	50.0	41.1

Aged 15+ years old

Supplementary Table 5a. Frequency of serotypes, stratified by year

VT	2006	2007	2008	2009	2010	2011	2012	2013	2014	2015	2016	2017	2018	Total
1	11	47	23	13	14	31	17	6	10	26	8	3	0	209
3	2	12	2	5	4	6	5	4	1	6	2	2	9	60
4	3	6	3	6	0	2	1	4	0	1	1	0	0	27
5	3	19	4	6	14	8	1	1	0	3	0	0	1	60
6A	0	3	2	1	1	2	1	2	1	1	3	1	1	19
6A/6B	0	0	0	1	2	0	0	1	0	0	0	0	0	4
6B	0	8	1	2	0	3	2	1	0	2	1	0	0	20
7F	2	6	3	4	2	3	1	1	0	1	0	0	2	25
9V	0	2	3	1	1	0	0	1	0	0	0	2	2	12
14	1	8	0	1	1	2	0	0	0	3	1	0	2	19
18C	0	3	0	1	1	0	0	1	1	0	1	0	0	8
19A	2	4	2	3	2	2	1	2	3	1	4	1	1	28
19F	0	1	0	0	1	2	0	1	0	0	0	0	0	5
23F	3	2	0	1	2	0	5	3	0	1	1	0	1	19
Total	27	121	43	45	45	61	34	28	16	45	22	9	19	515
NVT	19	54	39	43	36	32	35	34	19	24	36	29	26	426
Recovered	46	175	82	88	81	93	69	62	35	69	58	38	45	941
Unrecovered	6	27	57	31	11	6	3	3	3	6	0	0	3	156

Supplementary Table 5b. Prevalence of VT as portion of VT carriage, stratified by year

Serotype	2006	2007	2008	2009	2010	2011	2012	2013	2014	2015	2016	2017	2018	Total
1	40.7	38.8	53.5	28.9	31.1	50.8	50.0	21.4	62.5	57.8	36.4	33.3	0.0	40.6
3	7.4	9.9	4.7	11.1	8.9	9.8	14.7	14.3	6.3	13.3	9.1	22.2	47.4	11.7
4	11.1	5.0	7.0	13.3	0.0	3.3	2.9	14.3	0.0	2.2	4.5	0.0	0.0	5.2
5	11.1	15.7	9.3	13.3	31.1	13.1	2.9	3.6	0.0	6.7	0.0	0.0	5.3	11.7
6A	0.0	2.5	4.7	2.2	2.2	3.3	2.9	7.1	6.3	2.2	13.6	11.1	5.3	3.7
6A/6B	0.0	0.0	0.0	2.2	4.4	0.0	0.0	3.6	0.0	0.0	0.0	0.0	0.0	0.8
6B	0.0	6.6	2.3	4.4	0.0	4.9	5.9	3.6	0.0	4.4	4.5	0.0	0.0	3.9
7F	7.4	5.0	7.0	8.9	4.4	4.9	2.9	3.6	0.0	2.2	0.0	0.0	10.5	4.9
9V	0.0	1.7	7.0	2.2	2.2	0.0	0.0	3.6	0.0	0.0	0.0	22.2	10.5	2.3
14	3.7	6.6	0.0	2.2	2.2	3.3	0.0	0.0	0.0	6.7	4.5	0.0	10.5	3.7
18C	0.0	2.5	0.0	2.2	2.2	0.0	0.0	3.6	6.3	0.0	4.5	0.0	0.0	1.6
19A	7.4	3.3	4.7	6.7	4.4	3.3	2.9	7.1	18.8	2.2	18.2	11.1	5.3	5.4
19F	0.0	0.8	0.0	0.0	2.2	3.3	0.0	3.6	0.0	0.0	0.0	0.0	0.0	1.0
23F	11.1	1.7	0.0	2.2	4.4	0.0	14.7	10.7	0.0	2.2	4.5	0.0	5.3	3.7
**Total**	**100**	**100**	**100**	**100**	**100**	**100**	**100**	**100**	**100**	**100**	**100**	**100**	**100**	**100**

Supplementary Table 5c. Prevalence of VT as portion of total carriage, stratified by year

Serotype	2006	2007	2008	2009	2010	2011	2012	2013	2014	2015	2016	2017	2018	Total
1	23.9	26.9	28.0	14.8	17.3	33.3	24.6	9.7	28.6	37.7	13.8	7.9	0.0	22.2
3	4.3	6.9	2.4	5.7	4.9	6.5	7.2	6.5	2.9	8.7	3.4	5.3	20.0	6.4
4	6.5	3.4	3.7	6.8	0.0	2.2	1.4	6.5	0.0	1.4	1.7	0.0	0.0	2.9
5	6.5	10.9	4.9	6.8	17.3	8.6	1.4	1.6	0.0	4.3	0.0	0.0	2.2	6.4
6A	0.0	1.7	2.4	1.1	1.2	2.2	1.4	3.2	2.9	1.4	5.2	2.6	2.2	2.0
6A/6B	0.0	0.0	0.0	1.1	2.5	0.0	0.0	1.6	0.0	0.0	0.0	0.0	0.0	0.4
6B	0.0	4.6	1.2	2.3	0.0	3.2	2.9	1.6	0.0	2.9	1.7	0.0	0.0	2.1
7F	4.3	3.4	3.7	4.5	2.5	3.2	1.4	1.6	0.0	1.4	0.0	0.0	4.4	2.7
9V	0.0	1.1	3.7	1.1	1.2	0.0	0.0	1.6	0.0	0.0	0.0	5.3	4.4	1.3
14	2.2	4.6	0.0	1.1	1.2	2.2	0.0	0.0	0.0	4.3	1.7	0.0	4.4	2.0
18C	0.0	1.7	0.0	1.1	1.2	0.0	0.0	1.6	2.9	0.0	1.7	0.0	0.0	0.9
19A	4.3	2.3	2.4	3.4	2.5	2.2	1.4	3.2	8.6	1.4	6.9	2.6	2.2	3.0
19F	0.0	0.6	0.0	0.0	1.2	2.2	0.0	1.6	0.0	0.0	0.0	0.0	0.0	0.5
23F	6.5	1.1	0.0	1.1	2.5	0.0	7.2	4.8	0.0	1.4	1.7	0.0	2.2	2.0
**Total**	**58.7**	**69.1**	**52.4**	**51.1**	**55.6**	**65.6**	**49.3**	**45.2**	**45.7**	**65.2**	**37.9**	**23.7**	**42.2**	**54.7**

Supplementary Table 5d. Prevalence of NVT as portion of total carriage, stratified by year

	2006	2007	2008	2009	2010	2011	2012	2013	2014	2015	2016	2017	2018	Total
NVT	41.3	30.9	47.6	48.9	44.4	34.4	50.7	54.8	54.3	34.8	62.1	76.3	57.8	45.3

Supplementary Table 6. Frequency of VT- and NVT-IPD stratified by year*

	Infants			1-4 years old			5-14 years old			15+ years old Adolescents & adults			All ages		
**year**	**NVT**	**VT**	**Total**	**NVT**	**VT**	**Total**	**NVT**	**VT**	**Total**	**NVT**	**VT**	**Total**	**NVT**	**VT**	**Total**
2006	41	42	83	33	20	53	14	29	43	14	29	43	102	120	222
2007	30	44	74	10	28	38	11	25	36	11	25	36	62	122	184
2008	29	14	43	8	25	33	14	29	43	14	29	43	65	97	162
2009	17	11	28	8	23	31	18	21	39	18	21	39	61	76	137
2010	9	19	28	9	13	22	17	17	34	17	17	34	52	66	118
2011	24	26	50	9	27	36	18	53	71	18	53	71	69	159	228
2012	17	8	25	15	8	23	15	26	41	15	26	41	62	68	130
2013	5	4	9	8	4	12	9	1	10	9	1	10	31	10	41
2014	1	5	6	6	2	8	8	11	19	8	11	19	23	29	52
2015	6	6	12	3	9	12	4	11	15	4	11	15	17	37	54
2016	7	6	13	9	3	12	12	8	20	12	8	20	40	25	65
2017	8	0	8	5	1	6	10	5	15	10	5	15	33	11	44
2018	2	5	7	4	0	4	4	4	8	4	4	8	14	13	27
**Total**	**196**	**190**	**386**	**127**	**163**	**290**	**154**	**240**	**394**	**154**	**240**	**394**	**631**	**833**	**1464**

*VT and NVT corrected for proportion serotyped

Supplementary Table 7. Incidence rate ratio of IPD using empirically observed cases and controls, 1 January 2006 to 31 December 2018.

		2006–2011		2012–2013		2014–2018		2014–2018 vs 2006–2011		
Serotype	Age group	Cases*	Incidence†	Cases	Incidence	Cases	Incidence	IRR‡	95% CI	p-value
All IPD	Infants	306	226·6	34	62·0	45	29·1	0·54	0·29, 1·01	0·05
	Children 1–4yrs	213	39·4	35	15·9	42	6·8	0·58	0·30, 1·12	0·11
	Children 5–14yrs	265	27·3	51	13·4	76	6·7	0·26	0·12, 0·54	<0·001
	Persons ≥15yrs	902	36·7	178	18·8	266	10·0	0·53	0·29, 0·99	0·05
VT IPD	Infants	156	115·5	12	21·9	22	14·2	0·62	0·32, 1·22	0·17
	Children 1–4yrs	136	25·2	12	5·5	15	2·4	0·24	0·10, 0·58	0·002
	Children 5–14yrs	174	17·9	27	7·1	39	3·4	0·21	0·08, 0·56	0·002
	Persons ≥15yrs	539	21·9	85	9·0	121	4·5	0·54	0·23, 1·25	0·15
NVT IPD	Infants	150	111·1	22	40·1	24	15·5	0·53	0·23, 1·22	0·13
	Children 1–4yrs	77	14·3	23	10·5	27	4·4	1·78	0·64, 4·96	0·27
	Children 5–14yrs	92	9·5	24	6·3	38	3·4	0·31	0·10, 1·00	0·05
	Persons ≥15y	363	14·8	93	9·8	145	5·4	0·50	0·20, 1·26	0·14

* Cases of 13VT and non-13VT corrected for proportion serotyped. †Incidence per 100,000 age-specific population. ‡ Negative binomial regression of locally weighted incidence, adjusted for year. IPD=Invasive pneumococcal disease; IRR=Incidence Rate Ratio; CI=Confidence interval; 13VT=PCV13-valent vaccine serotypes
